# The association of family doctor contract service and patient trust in doctor: evidence from twenty-five village clinics of three counties in rural China

**DOI:** 10.1186/s12875-024-02298-4

**Published:** 2024-02-15

**Authors:** Linni Gu, Xiaoying Wang, Donghua Tian

**Affiliations:** 1 Health Management School, Inner Mongolia Medical University, Jinshan Development District, Huhehaote, 010110 China; 2 School of Social Development and Public Policy, Beijing Normal University, 19 Xinjiekou Wai Street, Haidian District, Beijing, 100875 China

**Keywords:** Contact service, Family doctor, Doctor-patient relationship, Trust

## Abstract

**Background:**

China is implementing the family doctor (FD) system to reform the primary healthcare (PHC). The family doctor contract service (FDCS) policy plays a crucial role in this system implementation, aiming to transform the doctor-patient relationship and enhance PHC quality. This study aims to investigate the impact of FDCS on the doctor-patient relationship in PHCs using field research methodology.

**Method:**

The field research methodology was employed to address the research questions. Quantitative methods were utilized for data collection and analysis. A structure questionnaire was used to collect data based on the research questions. Our investigation encompassed twenty-five village clinics across three counties in China. A total of 574 subjects helped us to finish this investigation in the study. The collected data was analyzed using statistical analysis including ordinary least squares (OLS) model and propensity scores matching model (PSM) to estimate the relationship.

**Result:**

The findings from ordinary least squares (OLS) regression revealed that FDCS had a positive influence on patient trust in doctors within PHCs, with patients who participated the FDCS exhibiting higher levels of trust compared to those who did not participate. Propensity score matching (PSM) analysis further confirmed these results by accounting for selection bias.

**Conclusions:**

The implementation of family doctor contract service has brought about significant transformation in the doctor-patient relationship within rural Chinese PHCs. In essence, it has revolutionized the service model of doctor in PHC, playing a pivotal role in improving primary health quality and enhance the service capability of doctors in PHC. This transformative process has been crucial for carrying out hierarchical diagnosis and treatment policy, which aims to adjust the medical service structure and optimizing the health service system. Therefore, it is imperative for government authorities and health administration departments to ensure continuous support for this essential service through appropriate formulation.

## Background

Family doctor (FD) system, as an important health policy measure to achieve the goal of primary health care, has experienced rapid development worldwide. It has been adopted by more than 50 countries and regions globally [1]. Furthermore, it is recognized by the World Health Organization (WHO) as “the most economical and accessible” form of healthcare [1, 2]. According to WHO report, more than 80% of illness can be effectively managed throughout primary health care service, with only 5% requiring specialized hospital treatment A well-implemented family doctor system has demonstrated positively related to health outcomes and medical cost containment [3]. The role of family doctor is university acknowledged as that of health gatekeeper who provides comprehensive health care services and necessary referrals when required.

The concept of the family doctor system originated in Western countries during the 18th century and was further developed in America in 1960s. In America, citizen have voluntary choice regarding registration with a family doctor service based on their preferences and medical insurance coverage [4, 5]. Conversely, In Britain’s National Health Service follows a national management model that mandates resident register with a designated family doctor [5]. In Germany, family doctor primarily provides outpatient service for local residents while serving as gatekeeper within health care system. Cuba established its family doctor system in 1980s which plays extremely important role in the three-tiered health system. Each family doctor along with a nurse serves approximately 120 families or around 600–800 residents providing preventive care, health education, and medical treatment for local communities.

However, in China, the concept of family doctor was firstly introduced in 1980s, and formally proposed by central government in 2003 to develop community health service in urban areas and township health center in rural area. In 2009, a new round of health reform was launched to establish primary health centers (PHCs) throughout China. The FD system was officially established within the PHC delivery system as part of health promotion efforts to improve residents’ well-being. In 2011, the family doctor system was become national strategy with publication of *Guiding Opinions of the State Council on the Establishment of the General Practitioner System*. In 2013, family doctor contract service (FDCS) was piloted in rural areas and fully implemented nationwide in 2016.

FDCS is a contractual service provided by family doctor along with nurse that ensure continuity, safety, and effective basic medical service based on agreements signed with local residents. These contract services were divided into three packages: one for basic public health service according to the public health provisions; another for basic package combined with personalized options; and an integrated medical treatment and nursing care package. The basic package is free for residents while fees ranging from RMB 50 Yuan to RMB 800 Yuan apply for personalized needs-based services. The aim of carrying out the FDCS by China’s government is to improve the quality of primary health, enhance the relationship between doctor and patient and transform the existing inverse triangle medical service structure caused by patients’ preference for visit specialist doctors in tier-2 and tier-3 hospitals regardless their illness. There is a deeply ingrained belief that tier-2 and tier-3 hospitals are equipped with high- quality doctors and advanced medical equipment. As a result, patients flock to these hospitals, leading to challenges in accessing medical care (*Kanbing Nan*) and exorbitant costs (*Kanbing Gui*).

To address doctor-patient relationship and enhance patient trust in primary health care, the government has recently prioritized this issue, however, little research has been conducted on this topic, making it worth exploring for both research and policy purposes. Recent studies in China have primarily focused on patients’ willingness to sign FDCS [6–8], as well as its effect on various aspect such as medical expense, satisfaction, service utilization, non-communicable disease management, etc. [2, 9, 10]. Additionally, numerous studies have explored patient trust in doctor from the perspective of the medical satisfaction levels, medical utilization rates, treatment outcomes, communication skills of doctors, etc. [2, 11–14]. Although some researchers have recognized the significance of establishing a stable relationship with family doctors, the specific relationship between FDCS and patient trust has not been explored until now. Therefore, the aim of this study is to explore the relationship between FDCS and patient trust doctor in rural primary health centers. This study will address the following specific questions: (1) Does FDCS policy influence patient trust in doctors in rural primary health center? (2) Does government implementation of FDCS increase the patient trust in doctors in the primary health center? (3) What are the differences regarding trust in doctors between those who have signed contract service compared with those who haven’t? Targeting with these research questions, we collected data from three counties: DF district, WF and YN County. DF is one of the earlies pilot cities practicing family doctor contract service in China. WF and YN County implemented FDCS in 2016 after DF.

## Methods

### Study setting

According to the pre-test results, a total of twenty-five village clinics were selected for carrying out this study and assisting in addressing the research questions. These twenty-five village clinics were distributed across in three counties in three provinces. The selection of these three counties took in to account variations in geographical position, economic development and primary health care services. DF County is situated in the eastern affluent Jiangsu Province with a GDP of RMB 64.75 billion Yuan in 2017. Geographically, it encompasses a vast flat area and serves as coastal city. On the other hand, YN County is upland region located in the western part of Shandong Province with a GDP of RMB 26.27 billion Yuan. Lastly, WF Tujia Autonomous County is positioned within mountainous western region of Hubei Province in central China and has a GDP of RMB 6.549 billion Yuan. DF was the pilot site for implementing family doctor contract services due to its successful track recode and commendable execution of this policy, which subsequently inspired other provinces’ health managers to learn from their experience. Following DF’s lead, YN and WF also carried out this policy.

### Data collection and participants

A multistage random sampling method was employed to select village clinics and patients for this study. Firstly, the province was selected based on geographic location, economic development and the feasibility of carrying out this investigation. Secondly, the county was selected considering the convenience and financial affordability. DF, WF and YN county were specifically selected due to our familiarity with the local health administrative officer who could provide assistances when needed. Thirdly, twenty-five village clinics were chosen with the guidance of administrative officers from these three counties. Fourthly, participants were chosen from a patient list provided by village doctors who had visited doctors in the past one year. All selected participants were convened in the meeting room of village clinic under supervision of qualified investigators via telephone to complete the survey. Lastly, a total of 625 participants joined this investigation by completing questionnaires after providing informed consent. As a token of appreciation, all participants received a towel gift as gratitude for their participation. In total, 574 participants completed the questionnaires resulting in a response rate of 92%. Four investigators from Beijing Normal University who had received professional training, assisted in completing this investigation.

### Data collection statement

The data was collected in accordance with the principal of Declaration of Helsinki. The privacy of participants was safeguarded, ensuring complete anonymity during data processing. Prior to conducting the investigation, informed consent was obtained from all participants. As a token of appreciation, each participant received a towel. This survey was approved by the Ethics Committee of the School of Social Development and Public Policy at Beijing Normal University (committee’s reference number is 2,017,001).

### Measurements

The self-compiled structure questionnaire was utilized with the assistance of participants to investigate the research questions. The questionnaire encompassed demographic characteristics, medical insurance information, drug usage perception, family doctor contract service, and patient trust scale. It was developed based on the research questions, study design, health policy, culture aspects of the study sites, target population, and findings from literature review.

A pre-test for the questionnaire was conducted in ten village clinics located in SY district, Beijing city. The selection of pre-test participants was facilitated by local health administrative officer and village doctors. A total of 50 pre-test participants assisted us to test the questionnaire. Lastly, we got a good reliability and validity using Cronbach’s α and factorial analysis, the value of reliability were presented in the following variables introduction.

### Control variables

The control variables were selected based on previous studies [8, 9, 14–16], encompassing demographic characteristics, medical insurance information, drug usage perception.

**Demographic characteristics** were measured in this study to investigate participants’ basic information including gender, identity (farmer or non-farmer according to Huko registration), marital status, education background and annual family income.

**Patient attitude towards medical insurance** was measured to observe their opinions regarding minimum payment of rural cooperative medical insurance (RCMI), and attitude toward medicine expenditure after the reimbursement of RCMI.

**Drug usage perception** was measured to observe patient attitude towards the effectiveness of drug. This measurement was necessary due to the implementation of China’s *National Basic Drug System* in health system where all health unites were required use drugs from the category of National Basic Drug that were centrally purchased by provincial health administrations. It is measured using the question “do you satisfy with the drug supply at village clinic? and satisfy with the efficacy of drug treatment?”

### Independent variable

**Family doctor contract service** was measured using dichotomized variable (“yes or no”). The question “Have you signed up with the contract service with village doctors?” Participants could respond with either “yes or no”.

### Dependent variable

**Patient trust** was measured using Chinese version of the Wake Forest Physician Trust Scale (WFPTS), which has been widely used in studies examining patient-doctor relationships. This modified scale consists of ten items measuring two dimensions: kindheartedness, and medical skills as indicators of patients trust doctors. Each item was scored using a 5-point Likert scale. The reliability of the scale has showed a value of Cronbach’s α of 0.89 for this scale, consistent with previous study.

### Statistical analysis

To explore the association between patient participation the FDCS and their trust in doctor, the following strategies of statistical analysis were employed: firstly, descriptive statistical analysis was utilized to analyze the basic information of the participants; secondly, *t*-tests were conducted to examine the differences in trust between patients who signed or did not sign FDCS. Thirdly, considering the dependent variable trust was a continuous variable, the ordinary least squares (OLS) regression statistical analysis, a commonly used regression prediction model, was used to predict the relevant relationship between FDCS and patient trust in doctor (see model 1), *Trust*_*i*_ in model 1 represents the level of patient trust in doctor for the *i*th member of the patients; FDCS represents the preference selected by the *i*th patients in the primary health service, and *X*_*2*_…*X*_*k*_ represent the other controlled variables, including demographic variables, patients’ health attitude towards medical insurance, feeling about drug use that may affect the patient trust in doctor, *β*_0_ is the intercept term, *β*_*1*_ is the correlation coefficient matrix indicating how FDCS impacts patient trust in doctors, *β*_*2*_…*β*_*k*_ are correlation coefficients how other control variables affect patient trust in doctor, and ε_*i*_ is a random error term. Fourthly, due to highly self-selective behavior among contract services which makes it difficult to identify rural resident who signed contract service but did not visit village clinic within one year prior to this study’s data collection period. Consequently, estimation results from OLS regression may be biased due to the selection deviation. To mitigate the potential selection bias issues caused by this situation, the Propensity Scores Matching model (PSM) was used to further analysis. Theoretically, the PSM model can reduce the confounding factor causing selective deviation caused and address the potential endogenous problem in the model [17]. The theoretical framework of matching estimation is counterfactual deduction model that identifies the samples within the control group who are similar to the samples in the treated groups, in order to match them with counterfactual individuals. Thus, the PSM model is conducive to reduce the biased and ensuring consistent estimation. The PSM involves four steps: firstly, calculating the propensity score using logit models; secondly, matching based on this score; thirdly, evaluating the balance after matching, and finally, calculating the average treatment effect (ATT). The caliper matching, radius matching, and kernel matching were adopted in this study. Specifically, model 2 presented the effect of average treatment (ATT) on patient trust in doctor. Y_1_ represents the average value of the explanatory variable when the samples from treated group receive the intervention, while Y_0_ represents the average value assuming they did not receive the intervention.

model 1 $$Trus{t_i} = {\beta _0} + {\beta _1}FDCS + {\beta _2}{X_{i2}}.{\rm{ }}.{\rm{ }}.{\beta _k}{X_{ik}} + {\varepsilon _i}$$

model 2 $${\rm{ATT = E}}\left( {{{\rm{Y}}_{\rm{1}}}{\rm{|p = 1}}} \right){\rm{ - E}}\left( {{{\rm{Y}}_{\rm{0}}}{\rm{|p = 1}}} \right)$$

## Results

### Participants characteristics

Table 1 presented the fundamental information of the 574 participants. As far as the gender, males slightly outnumbered female. A significant majority of participants were engaged in farming, accounted for 91.64% in all participants. Regarding age distribution, a major part of participants was in the 41–50 years old. With respect to education background, a considerable number of participants graduated from middle school, and more than one third participants completed primary school. Over half of participants’ annual family income ranged from RMB10,000 to RMB30,000. Concerning medical expenditure, the majority incurred cost below RMB1,000. Turning to the policy satisfaction, most participants express contentment with the health policy.

**Table 1:** Participants characteristics

Variables	N (574)	Percentage (%)
**Gender**		
Male	315	54.88
Female	259	45.12
**Identity**		
Farmer	526	91.64
Non-farmer	48	8.36
**Age**		
≤ 40	72	12.54
41–59	264	45.99
≥ 60	238	41.46
**Marital status**		
Married	519	90.42
Unmarried	12	2.09
Divorced or widowed	43	7.49
**Education**		
Primary school or below	184	32.06
Junior school	254	44.25
Senior high school or above	136	23.69
**Family income (Yuan)**		
≤ 9,999	173	30.14
10,000–29,999	290	50.52
≥ 30,000	111	19.34
**Attitude toward minimum payment of RCMI**		
Low	69	12.02
Suitable	267	46.52
High	238	41.46
**Attitude toward medicine expenditure after the RCMI**		
Decrease	329	57.32
Unchanged	125	21.78
Increase	120	20.91
**Satisfaction with drug supply of village clinic**		
Yes	384	66.9
No	190	33.1
**Satisfaction with drug treatment effect**		
Bad	19	3.31
Good	555	96.69
**Status of contract service**		
Signed	358	62.37
No	216	37.63

### Comparison of contract service on patient trust in doctor

Table 2 presented the differences between signed contract service group and non-signed contract service group in the three counties. The statistical analysis of *t*-test was conducted to analyze the difference of the two groups. The results showed that the difference was existed significantly between the two groups. The participants of contract service in DF and WF were more than that in YN. Moreover, a significant difference was observed in patient trust in doctor between the two groups (*P* < 0.001).

**Table 2:** Contract service characteristics and comparison of the two groups

	Signed			Not signed				
	Freq.	Percent	Mean	Freq.	Percent	Mean	Mean-diff.	t
Df	138	78.41		38	25.19			
YN	96	47.76		105	52.24			
WF	124	62.94		73	37.06			
Trust	358		3.59	216		3.02	-0.17***	-3.93

* 0.01 < *p* ≤ 0.05, ** 0.001 < *p* ≤ 0.01, *** *p* ≤ 0.001

### Family doctor contract service influences patient trust doctor

A basic OLS model was employed in the study to examine the relationship between contract service and patient trust in doctor. The results showed that contract service had a positive influence on patient trust in doctor (*P* < 0.001) (see Table 3).

**Table 3:** Results of OLS

Variables	Patient trust doctor		
	Coefficient	Std. Err.	*p*
Contract service	0.282	(0.075)	*P* < 0.001
Gender	0.010	(0.069)	
Identity	-0.250	(0.127)	*P* < 0.05
Age	-0.132	(0.054)	*P* < 0.05
Marriage	-0.043	(0.063)	
Education	0.034	(0.048)	
Annual income	0.100	(0.051)	
Reimbursement ration	-0.334	(0.075)	*P* < 0.001
Reimbursement procedure	-0.351	(0.076)	*P* < 0.001
Drug effect	0.231	(0.190)	
_cons	-0.123	(0.454)	
*N*	574		
*R* ^2^	0.225		

* 0.01 < *p* ≤ 0.05, ** 0.001 < *p* ≤ 0.01, *** *p* ≤ 0.001

### Selection bias: a further analysis based on the PSM method

An analysis of Table 3 revealed that FDCS significantly influence patient trust in doctor in rural primary health. However, there were obvious difference between patient who signed and did not signed contract service with respect to rural patient trust in doctor. Table 4 further reported the PSM results for the influence of contract service on the patient trust in doctor. Line 1 reported the ATT coefficient for caliper matching, which was found to be 0.178. Line 2 reported the ATT coefficient of 0.159 for radius matching, while line 3 reported the coefficient of 0.153 for kernel matching. These ATT coefficients were consistent with the regression coefficient of FDCS in Table 3, indicating that contract service increased patient trust in doctor in rural primary health settings.

**Table 4:** PSM results for the influence of contract service on patient trust doctor

	Matching types	Treated	Controlled	Difference	S.E.	*p*
Patient trust doctor	Caliper matching	3.599	3.420	0.178	0.059	< 0.001
	Radius matching	3.599	3.440	0.159	0.048	< 0.001
	Kernel matching	3.601	3.449	0.153	0.046	< 0.001

* 0.01 < *p* ≤ 0.05, ** 0.001 < *p* ≤ 0.01, *** *p* ≤ 0.001

Note: The treatment group was signed contract service, the control group was not signed contract service

## Discussion

In this study, we explored the relationship between family doctor contract service and patient trust in doctor in the primary health service using the field survey data. To our knowledge, this is the first study to examine the relationship between FDCS and patient trust in doctor. The results revealed that family doctor contract service significant influenced rural patient trust in doctor across the three counties.

Patient trust in doctor was an important topic in establishing and improving doctor-patient relationship in the health system. Previous studies have testified that a strong trust relationship between doctor and patient can improve the treatment outcomes, patient adherence, satisfaction level, and medical experiences [18–20]. However, in China, especially in rural primary health, the trust relationship of patient to doctor were often challenged due to limitation in service capabilities and quality of care provided at the primary health centers. Patients tend to had less confidence in primary health providers compared with those worked at larger hospitals with more advanced medical resources available. Consequently, patient preferred to seeking medical attention from big hospital regardless their illness severity or nature (even for minor ailments like colds or stomachaches), leading to overcrowding issues at tier-2 and tier-3 hospitals. Therefore, it is imperative to increasing patients trust in doctors in primary health center as an urgent solution for alleviating overcrowding problems at big hospitals.

The results of Table 2 presented that a large number of patients who have signed the FDCS in this study. This high participation rate can be contributed to the widespread of the FDCS policy throughout China [21], as well as patients’ desire for health consultants to assist them with managing and supervising their health. However, some residents did not sign the family doctor contract service due to its voluntary nature, and insufficient advocacy of FDCS policy in rural area [9]. Notably, the number of signed contract service was higher in DF than WF and YN (78.41%, 62.94%, 47.76%), as DF is the pilot city with effective publicity and execution of this policy.

The results of OLS regression and PSM analysis found that family doctor contract service significantly influenced patient trust in doctor in the primary health of rural China (*β* = 0.282, *P* < 0.001), which explained that the family doctor contract service was conducive to transform doctor-patient relationship, attract patients and improve patients retention rates in the primary health care in rural area. The reasons for this transformation brought by FDCS were that FDCS provided personalized needing health service such as free illness consultations, follow-up care, home sick-bed, etc., which catered to individual patients demand and encourage seeking medical care outside hospitals. Meanwhile, the free basic public health services provided by the primary health doctor can alleviate patient medical expenditure, while promoting health protection and disease prevention [3, 5]. Additionally, the FDCS can increase the adherence of patient to doctor through complimentary body examination including blood pressure and blood glucose monitoring. In comparison, prior to carrying out the FDCS, doctors in primary health centers played a role as a drug seller who profited from selling medications for minor ailments. This led rural patients to question their medical skills and service quality, consequently prompt them to prefer seeking medical treatment independently without guidance from primary health care providers.

In this study, family doctor contract service influenced patient trust in doctor was firstly explored. Although numerous studies have discussed the trust relationship between patient and doctor, this study mainly focused on understanding this relationship from the perspective of patient. Recent studies has highlighted that doctor’s effective communication skills were the crucial in influencing patient trust in doctors, emphasizing the need for doctors to acquire and enhance their communication skills [22–24]. Earlier studies have also identified service quality as a significant factor affecting patient trust in doctor, suggesting that health service provider should prioritize improving doctor’s medical skills, health equipment and overall health service capabilities [25–27]. Furthermore, other studies have found that factors such as family income, social media usage and health policy reforms can influence patient-doctor trust relationship [28, 29]. This study provides additional evidence, FDCS, on the impact of patient trust in doctor in primary health care, thereby contributing to the growing body of literature examining the relationship between family doctor contract service and patient trust in China. As highlighted above, FDCS has demonstrated its ability to influence patients’ medical seeking behavior and health-related behavior while also increased their adherence to primary health providers within the Chinese healthcare system. Based on this, the implementation of FDCS has successfully achieved its objective of transforming both patient medical seeking behaviour and overall structure of China’s health care system.

## Conclusion

To the best of our knowledge, this study represents first exploration of the relationship between contract service and patient trust in doctor in primary health of rural China. The implementation of family doctor contract service played a pivotal role in transforming doctor-patient relationship, thereby addressing issues related to medical seeking behavior and attracting more patients to seek care at primary health centers. More importantly, this service is crucial for facilitating the successful implementation of China’s hierarchical diagnosis and treatment policy. Therefore, we recommended that the government and health administration department ensure the provision of this service by formulating appropriate regulations that safeguard both patient and doctor rights.

### Limitations

Some limitations should be acknowledged in this study. Firstly, this study was conducted in three counties in primary health service of rural China, which the small sample size cannot be popularized to the whole country. Secondly, while we adjusted some potential confounders in multiple regression models, there may still exist other potential confounders. Thirdly, this study only analyzed the relationship between FDCS and patient trust in doctor, the mechanism and pathway between them cannot be analyzed in this study. Future research will delve into these aspects.

## Data Availability

The data set is available from the corresponding author, who will provide it on reasonable request.

## Abbreviations

FDCS: Family doctor contract service
GP: General practitioner
OLS: Ordinary least squares
PHC: Primary healthcare center
PSM: Propensity score matching
RCMI: Rural cooperative medical insurance
WFPTS: Wake Forest Physician Trust Scale
